# Supplementary material for: Polyketide synthase gene clusters in the ericoid mycorrhizal fungus Oidiodendron maius: genome-wide prediction, expression profiling, and functional characterization of knockout mutants
Source: Front Plant Sci. 2026 Sep 16;17:1924430. doi: 10.3389/fpls.2026.1924430 (PMC13623903; doi:10.3389/fpls.2026.1924430)
Supplement: Supplementary file 1 [file SupplementaryFile1.zip › Supplementary Materials/Supplementary Folder 1.zip!/Oidma1_AssemblyScaffolds_Repeatmasked/knownclusterblast/cluster50/jgi.p_Oidma1_45984_mibig_hits.html]

| MIBiG Protein | Description | MIBiG Cluster | MiBiG Product | % ID | % Coverage | BLAST Score | E-value |
| --- | --- | --- | --- | --- | --- | --- | --- |
| BAC20568.1 | efflux\_pump | BGC0000039 | Compactin\_biosynthetic\_gene\_cluster | 45 | 96.4879852126 | 420 | 3.2e-117 |
| AAD34558.1 | unknown | BGC0000089 | Lovastatin\_biosynthetic\_gene\_cluster | 44 | 96.4879852126 | 399 | 4.5e-111 |
| ABA02247.1 | efflux\_pump | BGC0000098 | Monacolin\_K\_biosynthetic\_gene\_cluster | 44 | 93.1608133087 | 394 | 1.9e-109 |
| CAP96439.1 |  | BGC0000420 | Roquefortine\_C\_/\_meleagrin\_biosynthetic\_gene\_cluster | 38 | 91.8669131238 | 354 | 2.2e-97 |
| AMR44276.1 | MFS\_multidrug\_transporter | BGC0001398\_c3 | Phomopsins\_biosynthetic\_gene\_cluster | 40 | 96.8576709797 | 351 | 1.8e-96 |
| AAS90069.1 | AflT | BGC0000010 | Aflatoxin\_biosynthetic\_gene\_cluster | 38 | 96.303142329 | 342 | 8.5e-94 |
| CBF87265.1 | MFS\_toxin\_efflux\_pump\_(AflT),\_putative\_(AFU\_orthologue;\_AFUA\_1G12620) | BGC0000680\_c2 | Austinol/dehydroaustinol,\_locus\_1\_biosynthetic\_gene\_cluster | 38 | 96.6728280961 | 340 | 3.2e-93 |
| ADY16699.1 | TqaJ | BGC0001142 | Tryptoquialanine\_biosynthetic\_gene\_cluster | 39 | 90.7578558226 | 333 | 3.9e-91 |
| AAS89998.1 | AflT | BGC0000007 | Aflatoxin\_biosynthetic\_gene\_cluster | 37 | 96.303142329 | 333 | 3.9e-91 |
| BAD29973.1 | transporter\_protein | BGC0000676 | Aphidicolin\_biosynthetic\_gene\_cluster | 36 | 96.4879852126 | 332 | 8.8e-91 |
| AAS90021.1 | AflT | BGC0000008 | Aflatoxin\_biosynthetic\_gene\_cluster | 37 | 96.303142329 | 330 | 3.3e-90 |
| AAS90092.1 | AflT | BGC0000006 | Aflatoxin\_biosynthetic\_gene\_cluster | 37 | 96.303142329 | 329 | 5.7e-90 |
| AAS90046.1 | AflT | BGC0000009 | Aflatoxin\_biosynthetic\_gene\_cluster | 37 | 96.303142329 | 329 | 9.7e-90 |
| BAE71313.1 | putative\_ABC\_transporter | BGC0000004 | Aflatoxin\_biosynthetic\_gene\_cluster | 39 | 86.1367837338 | 320 | 4.5e-87 |
| ACD39756.1 | major\_facilitator\_superfamily\_transporter | BGC0000076 | Hypothemycin\_biosynthetic\_gene\_cluster | 37 | 97.7818853974 | 316 | 5e-86 |
| ACD39765.1 | major\_facilitator\_superfamily\_transporter | BGC0000077 | Hypothemycin\_biosynthetic\_gene\_cluster | 37 | 97.7818853974 | 316 | 5e-86 |
| XP\_002384861.1 | MFS\_multidrug\_transporter,\_putative | BGC0001234\_c2 | 4,4'-piperazine-2,5-diyldimethyl-bis-phenol\_biosynthetic\_gene | 36 | 85.7670979667 | 305 | 1.1e-82 |
| ADI24949.1 | GsfJ | BGC0000070 | Griseofulvin\_biosynthetic\_gene\_cluster | 37 | 94.4547134935 | 297 | 2.4e-80 |
| EAQ86390.1 | hypothetical\_protein | BGC0001405 | Chaetoviridin\_/\_chaetomugilin\_biosynthetic\_gene\_cluster | 37 | 81.7005545287 | 295 | 1.6e-79 |
| CCE28984.1 | probable\_DHA14-like\_major\_facilitator;\_ABC\_transporter | BGC0001365 | Clapurines\_biosynthetic\_gene\_cluster | 32 | 96.6728280961 | 284 | 3.6e-76 |
| ADM34144.1 | efflux\_pump | BGC0001084 | Notoamide\_/\_stephacidin\_biosynthetic\_gene\_cluster | 32 | 92.0517560074 | 267 | 2.7e-71 |
| EAA65599.1 | hypothetical\_protein | BGC0000022 | Asperfuranone\_biosynthetic\_gene\_cluster | 33 | 98.3364140481 | 265 | 1.7e-70 |
| ACZ66257.1 | APS11 | BGC0000304 | Apicidin\_biosynthetic\_gene\_cluster | 34 | 89.8336414048 | 260 | 5.5e-69 |
| AHV78251.1 | ResE | BGC0001246 | Trans-resorcylide\_biosynthetic\_gene\_cluster | 31 | 93.9001848429 | 253 | 4e-67 |
| AAB36607.1 | toxin\_pump | BGC0000370\_c2 | HC-toxin\_biosynthetic\_gene\_cluster | 31 | 94.26987061 | 252 | 1.2e-66 |
| AGN71625.1 | putative\_HC-toxin\_efflux\_carrier\_TOXA | BGC0000027 | Azaphilone\_biosynthetic\_gene\_cluster | 39 | 58.9648798521 | 245 | 1.4e-64 |
| CBF76046.1 | conserved\_hypothetical\_protein | BGC0001399 | Fellutamide\_B\_biosynthetic\_gene\_cluster | 32 | 84.4731977819 | 241 | 2.7e-63 |
| EAU36749.1 | predicted\_protein | BGC0000292 | Acetylaranotin\_biosynthetic\_gene\_cluster | 33 | 87.8003696858 | 233 | 4.2e-61 |
| ctg1\_orf0002 |  | BGC0000688 | Copalyl\_diphosphate\_biosynthetic\_gene\_cluster | 33 | 71.1645101664 | 219 | 1.1e-56 |
| ACS68556.1 | major\_facilitator\_superfamily\_protein | BGC0001026 | NG-391\_biosynthetic\_gene\_cluster | 30 | 85.0277264325 | 178 | 2.8e-44 |
| CAF31575.1 | putative\_kanamycin\_efflux\_protein | BGC0000705 | Kanamycin\_biosynthetic\_gene\_cluster | 29 | 91.8669131238 | 167 | 3.7e-41 |
| CAF60521.1 | putative\_efflux\_protein | BGC0000704 | Kanamycin\_biosynthetic\_gene\_cluster | 29 | 91.8669131238 | 167 | 3.7e-41 |
| BAE56594.1 |  | BGC0001123 | Aspirochlorine\_biosynthetic\_gene\_cluster | 29 | 75.7855822551 | 163 | 7.1e-40 |
| AAF00219.1 | transporter | BGC0000277 | Urdamycin\_biosynthetic\_gene\_cluster | 26 | 90.9426987061 | 158 | 2.3e-38 |
| AMY15055.1 | MFS\_transporter | BGC0001339 | Squalestatin\_S1\_biosynthetic\_gene\_cluster | 24 | 91.4972273567 | 154 | 4.3e-37 |
| AGA37273.1 | efflux\_pump | BGC0000819 | Paraherquamide\_biosynthetic\_gene\_cluster | 37 | 32.9020332717 | 117 | 5.8e-26 |
| AAZ77686.1 | ChlG | BGC0000036 | Chlorothricin\_biosynthetic\_gene\_cluster | 26 | 70.6099815157 | 111 | 4.2e-24 |
| AGN74873.1 | major\_facilitator\_transporter | BGC0000459 | Griseoviridin\_/\_viridogrisein\_biosynthetic\_gene\_cluster | 24 | 94.8243992606 | 74 | 3.3e-13 |
| BAM73629.1 | putative\_bagremycin\_transporter | BGC0000889 | Bagremycin\_biosynthetic\_gene\_cluster | 24 | 53.6044362292 | 60 | 8.4e-09 |
